# Cigalike electronic nicotine delivery systems e-liquids contain variable levels of metals

**DOI:** 10.1038/s41598-020-67789-7

**Published:** 2020-07-17

**Authors:** Heather M. Neu, Angela Lee, Joel E. P. Brandis, Vyomesh Patel, Abraham Schneider, Maureen A. Kane, Richard N. Dalby, Sarah L. J. Michel

**Affiliations:** 1 Department of Pharmaceutical Sciences, University of Maryland School of Pharmacy, Baltimore, MD USA; 2 Center for Tobacco Products, US Food and Drug Administration, Silver Spring, MD USA; 3 Department of Oncology and Diagnostic Sciences, University of Maryland School of Dentistry, Baltimore, MD USA

**Keywords:** Environmental impact, Risk factors, Bioanalytical chemistry, Mass spectrometry

## Abstract

Electronic nicotine delivery systems (ENDS) are prefilled, battery-operated products intended to deliver nicotine to the user via an inhaled complex aerosol formed by heating a liquid composed of propylene glycol and glycerol, also referred to as vegetable glycerin and collectively called e-liquid, that contains nicotine and various flavor ingredients. Since their introduction in 2006, the number of ENDS on the market has increased exponentially. Despite their growing ubiquity, the possible health risks associated with ENDS use remain poorly understood. One potential concern is the presence of toxic metals in the e-liquid and aerosol. Herein, we report the evaluation of the metal content in the e-liquids from a series of commercially available cigalike ENDS brands (various flavors) determined using inductively coupled plasma mass spectrometry (ICP-MS) following e-liquid extraction. Each brand of cigalike ENDS was purchased at least three times at retail outlets in the Baltimore, Maryland metropolitan region over a period of six months (September 2017 to February 2018). This allowed for comparison of batch-to-batch variability. Several potentially toxic metals, including lead, chromium, copper, and nickel were detected in the e-liquids. In addition, high variability in metal concentrations within and between brands and flavors was observed . The internal assembled parts of each cartridge were analyzed by X-ray imaging, before dissembling so that the materials used to manufacture each cartridge could be evaluated to determine the metals they contained. Following washing to remove traces of e-liquid, lead, chromium, copper and nickel were all detected in the cigalike ENDS prefilled cartridges, suggesting one potential source for the metals found in the e-liquids. Collectively, these findings can inform further evaluation of product design and manufacturing processes, including quantification of metal concentrations in e-liquids over foreseeable storage times, safeguards against high concentrations of metals in the e-liquid before and after aerosolization (by contact with a metal heating coil), and control over batch-to-batch variability.

## Introduction

Cigalike electronic cigarettes (e-cigarettes) are a subset of electronic nicotine delivery systems (ENDS). They are battery-operated and intended to deliver nicotine to the user via an inhaled aerosol formed by heating an e-liquid prefilled into a cartridge. The e-liquid typically has a base formulation comprised of propylene glycol, vegetable glycerin, flavorings, and nicotine^1,2^. ENDS, that may be perceived as less harmful than combustible cigarette smoking, were initially developed as an alternative to cigarettes and targeted current tobacco smokers^3^. In recent years, the number of ENDS, designs, and flavors available has expanded rapidly. There are now several hundreds of ENDS products on the market, with a recent study noting that ~ 430 different brands were available in 2017^4^, and this category of tobacco product is projected to become a $61.4 billion business by 2025^5^. Concomitantly, the demographic of ENDS users has shifted towards teens and young adults^6^. There is a growing concern of nicotine addiction among youth and for the use of ENDS to serve as a potential gateway for other tobacco products such as cigarettes^7^. In addition, reports of potential toxicity associated with ENDS are emerging, suggesting that these products are not harmless^8–10^. The U.S. Food and Drug Administration (FDA) regulates ENDS and other tobacco products, under the Family Smoking Prevention and Tobacco Control Act. Research into the toxicity and adverse long-term effects from ENDS use can inform regulatory decision-making.

There are limited toxicological data available for the inhaled ingredients present in e-liquids and the aerosols generated by their use. There have been a few reports characterizing the constituents of aerosols generated from different ENDS. Both potentially toxic volatile organic molecules (likely byproducts of e-liquid heating or degradation) and, potentially toxic metals were observed in the aerosols studied^11–14^. The source of the metals detected has not been clearly established^12–20^. In one study on user-fillable tank type ENDS, the e-liquid and the ENDS components that the e-liquid is in contact with during storage and subsequent aerosolization were found to be the source of metals^13,15^. Whereas, in another study of user-filled tank type ENDS, the metals in the e-liquid did not transfer to the aerosol^21^. These differences may reflect the differences in products investigated and/or the puffing regimes utilized. Whether the metals detected in ENDS generated aerosols are derived from the e-liquid and the ENDS hardware remains an open question, especially for disposable products for which there is published evidence for the presence of metals in the aerosol^13^, but limited data are available for the e-liquids.

Herein, we sought to develop and implement an experimental protocol to identify the metal content in a series of commercially available disposable ENDS. As part of our approach, we focused on evaluating the batch to batch variability within products, as there are limited published data concerning quality control of ENDS products. The key findings of our studies include the presence of metals known to be toxic at elevated concentrations in the e-liquids and significant batch to batch variability of these metals, suggesting limited quality control. Moreover, we detected some of the same metals in the cartridge hardware that contains and aerosolizes the e-liquid, suggesting that the hardware may be a source of metals. We also provide images of ENDS internal assembled structure obtained via X-ray methods. To our knowledge, these are the first X-ray images of ENDS and they provide insight into the contact points between the e-liquid and the cartridge.

## Results

### Products surveyed

Our goal was to analyze cigalike ENDS that had been stored under retail or commercial conditions, so as to study the products as used by consumers. A series of purchases of ENDS were made from geographically separated gas stations and convenience stores in the Greater Baltimore Metropolitan Region (Baltimore City, Baltimore County, and Howard County) between September 2017 and February 2018. The cigalike ENDS obtained during the initial purchase (n =  > 3) were guided by anecdotal input from retailers regarding product popularity and are supported by published market data: VUSE was the most purchased product during this time period, with blu close behind^22^. During later visits to the same retail locations, the same brands and flavors were re-purchased. Table 1 lists these products and, their lot numbers and their expiration dates, if provided. Only MarkTen products displayed an expiration date. After purchase, all products were stored at controlled room temperature (22 ± 2 °C).

**Table 1:** List of cigalike ENDS brands, flavors and lot numbers purchased for evaluation (When provided on the product label the *do not use after date* is noted in parenthesis).

Brand	Flavor	Lot Number (“Do not use after” Date)			
		Purchase 1	Purchase 2	Purchase 3	Purchase 4
blu^b^	Classic tobacco	701613565317	5T05132407919	7A0613487057	6E23134555455
	Carolina bold	6A23133211433	6A17133211612	6A2513329346	^a^N/A
	Magnificent menthol	7R0313564461	7H211359656	7I121362947	6E05134157117
	Cherry crush	7C05134935511	7A1013493552	7H191359659	7A1013493552
MarkTen	Classic	BS2117B (2/12/18)	BS16179 (1/8/18)	BS42171 (7/9/18)	BS42171 (7/9/18)
	Menthol	BS27176 (3/26/18)	BS34173 (5/18/18)	BS42173 (7/9/18)	BS42173 (7/9/18)
	Summer fusion	BS22177 (2/12/18)	BS22177 (2/12/18)	BS22171 (2/12/18)	BS2117N (2/12/18)
	Winter mint	BS2117M (2/12/18)	BS2117M (2/12/18)	BS38176 (6/11/18)	BS2117M (2/12/18)
Vuse Solo^b^	Original	KV005KF7	KQ006SG7	KQ006LM7	KE008ML7
	Menthol	KN0065G7	KT0063H7	KR006HL7	KT006TM7
	Mint	JF006NF7	J0006JK6	JD006FJ7	N/A
	Berry	JE006NL6	JP0061D7	JE006FA7	JK006FA7
	Chai	JI005TM6	JM0052L6	JV005IB7	JI005TM6
	Crema	JH006SE7	JL006ND7	JB006MC7	KB005XK6
Vuse Vibe^b^	Original	O| 100SF7	O| 100XE7	O| 100GK7	O| 100QE7
	Menthol	N/A^a^	O| 100TB7	N/A	O| 100VC7
	Nectar	O| 100HE7	O| 100YM6	O| 100EJ7	O| 100VD7
	Melon	O| 1002M6	O| 100PA7	O| 100VJ7	O| 100PA7
Blank	Propylene glycol	BCBS5606V			
	Glycerol	STBG7230			

^a^N/A indicates the brand and flavor combination was unavailable during the purchase attempt.

^b^Neither the cartridge nor the packaging mentioned a *do not use after* date.

### E-liquid sample collection

**Figure 1:**
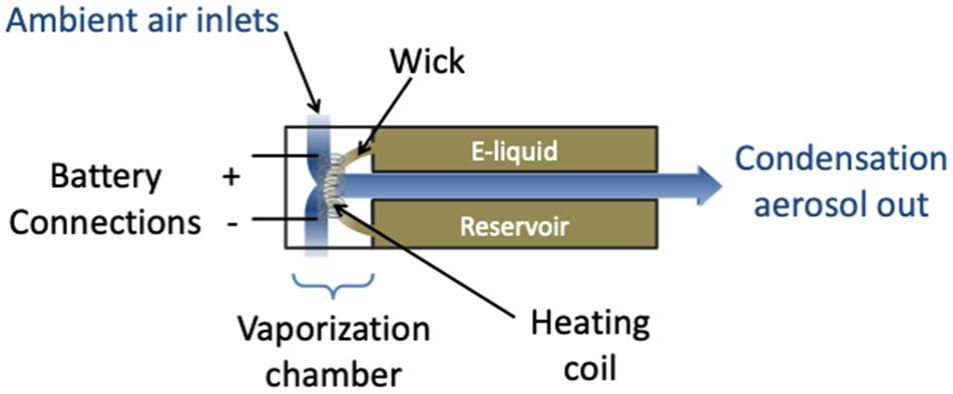
Schematic representation of a typical ENDS cartridge prefilled with e-liquid.

**Figure 2:**
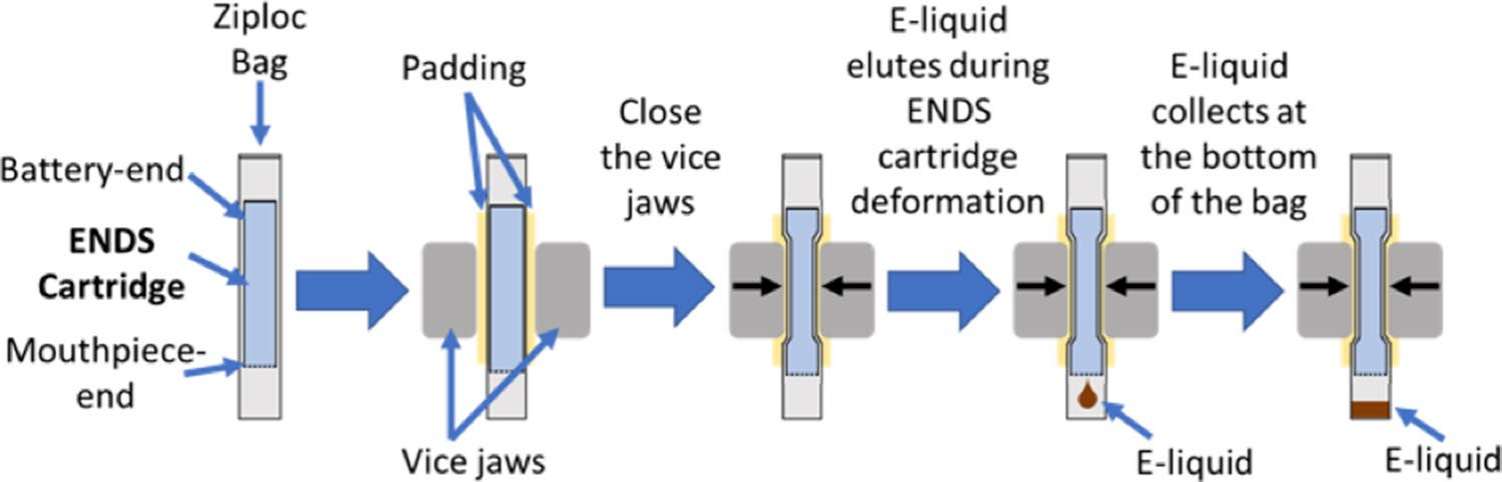
Diagram of e-liquid extraction protocol. The ENDS cartridge, placed in a Ziploc bag, is deformed via vice jaws leading to collection of e-liquid at the bottom of the Ziploc bag.

The general architecture of an ENDS cartridge includes an aerosolization chamber, a metal heating coil and an e-liquid reservoir (Fig. 1). To date, there are no established methods for e-liquid extraction from manufactured closed-filled ENDS cartridges. Aware that centrifugation does not always result in liquid removal and of the ubiquity of metals in the environment, and specifically on labware, and in metal pliers, drills and cutting tools, we developed and optimized a simple, high-throughput alternative sampling approach. This involved using a vice to distort cigalike ENDS prefilled cartridges to the point of rupture within metal-free single use plastic bags which were handled with gloved hands, then collecting the e-liquid with minimal exposure to laboratory work surfaces, as shown in Fig. 2. We limited the time e-liquids were in contact with new surfaces exposed during canister rupture to less than 30 min. This approach allowed us to collect samples of the e-liquid that we could accurately weigh and analyze for metal content via Inductively Coupled Plasma Mass Spectrometry (ICP-MS).

### Metals detected by ICP-MS

The metal content of the e-liquids that were obtained from the sampling was measured via ICP-MS. ICP-MS provides a quantitative measure of metal concentrations with detection limits as low a part per billion, and it is widely used, for example it is the gold standard for measurement of metal impurities in pharmaceuticals^23^. Our protocol involved initially running a ‘semi-quantitative analysis’ via ICP-MS which provides a rapid screen to identify all metals present in each e-liquid. The metals that were found to be present at elevated levels via semi-quantitative analysis were then re-measured using quantitative analysis to accurately determine their concentrations. These metals were lead (Pb), chromium (Cr), copper (Cu), magnesium (Mg), iron (Fe), zinc (Zn) and cobalt (Co). Propylene glycol (PG) and vegetable glycerin (VG, glycerol) did not contain any measurable levels of metals. The method was validated per FDA Guidance for Industry (issued by the Center for Drug Evaluation and Research) as described in Supplementary Information^24^. Although this guidance represents the FDA’s current thinking regarding bioanalytical method validation information for sponsors of investigational new drug applications (INDs) or applicants of new drug applications (NDAs), abbreviated new drug applications (ANDAs), biologic license applications (BLAs), and supplements, it was applicable to the method used here.

To this end, evaluation of potential harm from exposures to metals may be informed by comparisons with publicly available methods or reference limits. For example, in the case of drug products, pharmacopoeias such as the United States Pharmacopeia (USP) often fulfill this role. Given that there are not currently established methods or reference values for metals in e-liquids or ENDS aerosols, comparisons to USP limits provide an initial context for metal levels, albeit with those relevant for drug products, which have a risk–benefit tradeoff that is not applicable for tobacco products.

#### Metals in e-liquids: lead (Pb)

The ICP-MS data for Pb in the e-liquid of the products purchased are presented in Table 2. All data are shown in μg of lead per g of e-liquid. The USP limits metals (calling them ‘elemental impurities’) in drug products, and for Pb, the upper limit is 0.2 μg/g inhalation concentration and 0.5 μg/g oral concentration. All samples for which the mean Pb concentration equals or exceeds 0.2 μg/g are shown in bold in Table 2. These values ranged from 0.27 ± 0.03 to 290 ± 50 μg/g and included multiple blu and Vuse Vibe brand products. Of particular note were blu ‘Magnificent Menthol’ flavor and Vuse Vibe ‘Melon’ flavors, where Pb levels were measured at concentrations three orders of magnitude higher than the USP limit. Levels of Pb differed between each sampling purchase (most of which corresponded to a unique batch number), suggesting that rigorous quality control is not being applied to e-liquids and/or their containment system.

**Table 2:** Concentration of lead (Pb) in µg/g of e-liquid for the purchased cigalike ENDS cartridges.

Brand	Flavor	Purchases				
		Lead µg/g				
		1	2	3	4	Average
Blu	Classic tobacco	**0.27 (0.03)** ^a,b^	**0.87 (0.04)**	**18 (1)**	**1.2 (0.2)**	**5.1 (8)**
	Carolina bold	BLQ ^d^	**2.1 (0.6)**	0.15 (0.01)	N/A ^c^	**1.3 (1)**
	Magnificent menthol	**180 (20)**	**47 (30)**	**23 (1)**	**5.3 (0.5)**	**62 (70)**
	Cherry crush	0.24 (0.01)	N/A	22 (0.7)	0.65 (0.05)	**8.5 (10)**
MarkTen	Classic	BLQ	BLQ	BLQ	BLQ	UTD ^e^
	Menthol	BLQ	BLQ	BLQ	BLQ	UTD
	Summer fusion	BLQ	BLQ	BLQ	BLQ	UTD
	Winter mint	BLQ	BLQ	BLQ	BLQ	UTD
Vuse Solo	Original	BLQ	BLQ	0.01 (0.00)	BLQ	0.01 (0.00)
	Menthol	BLQ	BLQ	0.01 (0.00)	BLQ	0.01 (0.00)
	Mint	BLQ	BLQ	0.02 (0.00)	N/A	0.02 (0.00)
	Berry	BLQ	BLQ	0.03 (0.01)	BLQ	0.03 (0.00)
	Chai	BLQ	BLQ	BLQ	BLQ	UTD
	Crema	BLQ	BLQ	BLQ	BLQ	UTD
Vuse Vibe	Original	BLQ	BLQ	**11 (1)**	**1.1 (0.5)**	**5.9 (5)**
	Menthol	N/A	BLQ	N/A	**61 (0.4)**	**61 (0.4)**
	Nectar	0.14 (0.00)	BLQ	BLQ	**21 (3)**	**10 (10)**
	Melon	BLD	**1.0 (0.8)**	**290 (50)**	**97 (100)**	**130 (100)**
Blank	Propylene glycol	BLQ				
	Glycerol	BLQ				

All samples for which the mean lead concentration is equal to or exceeds the USP limits for lead in inhaled drug products (0.2 μg/g) are bolded.

^a^Concentrations are reported as mean (standard deviation), n = 3.

^b^Bold indicates the mean metal concentration exceeded the USP limit for an inhaled product.

^c^N/A indicates the brand and flavor combination was unavailable during the purchase attempt.

^d^Below lower limit of quantitation (BLQ).

^e^Unable to determine (UTD).

#### Metals in e-liquids: chromium (Cr)

The ICP-MS data for Cr in the products purchased are presented in Table 3 using the same approach described above for Pb. The USP upper limit for Cr is 0.3 μg/g inhalation concentration and 1,100 μg/g oral concentration. Levels of Cr that exceeded the USP limit ranged from 0.31 ± 0.01 to 1.2 ± 0.05 μg/g and were measured in blu and Vuse Solo products. Just as we observed for lead, the levels of Cr again varied amongst the purchases.

**Table 3:** Concentration of chromium (Cr) in µg/g of e-liquid for the purchased cigalike ENDS cartridges.

Brand	Flavor	Purchases				
		Chromium µg/g				
		1	2	3	4	Average
Blu	Classic Tobacco	**0.41 (0.03)**^a,b^	**0.65 (0.03)**	0.20 (*)	**0.47 (0.04)**	**0.49 (0.2)**
	Carolina Bold	BLQ^d^	BLQ	BLQ	N/A^c^	UTD^e^
	Magnificent Menthol	**0.55 (0.01)**	BLQ	BLQ	0.22 (0.00)	**0.42 (0.2)**
	Cherry Crush	0.22 (0.01)	N/A	BLQ	**0.35 (0.03)**	0.29 (0.07)
Mark Ten	Classic	0.23 (0.01)	0.24 (0.01)	0.18 (*)	BLQ	0.22 (0.02)
	Menthol	BLQ	BLQ	BLQ	BLQ	UTD
	Summer Fusion	0.33 (*)	0.24 (*)	0.25 (*)	BLQ	0.27 (0.05)
	Winter Mint	BLQ	BLQ	BLQ	BLQ	UTD
Vuse Solo	Original	BLQ	BLQ	BLQ	BLQ	UTD
	Menthol	BLQ	BLQ	BLQ	BLQ	UTD
	Mint	**1.0 (0.3)**	0.25 (0.00)	BLQ	N/A	**0.70 (0.5)**
	Berry	**0.45 (0.01)**	**0.58 (0.02)**	0.42 (*)	**0.31 (0.01)**	**0.45 (0.1)**
	Chai	**0.59 (0.1)**	0.22 (*)	0.29 (*)	**0.50 (0.1)**	**0.45 (0.2)**
	Crema	**0.50 (0.02)**	**1.21 (0.05)**	**0.52 (0.04)**	BLQ	**0.68 (0.3)**
Vuse Vibe	Original	BLQ	BLQ	BLQ	BLQ	UTD
	Menthol	N/A	BLQ	N/A	BLQ	UTD
	Nectar	BLQ	BLQ	BLQ	BLQ	UTD
	Melon	BLQ	BLQ	0.18 (*)	BLQ	0.18 (*)
Blank	Propylene Glycol	BLQ				
	Glycerol	BLQ				

All samples for which the mean chromium concentration is equal to or exceeds the USP limits for Cr (0.3 μg/g) are bolded.

^a^Concentrations are reported as mean (standard deviation), n = 3 unless otherwise noted by (*).

^b^Bold indicates the mean chromium concentration exceeded the USP limit for an inhaled product.

^c^N/A indicates the brand and flavor combination was unavailable during the purchase attempt.

^d^Below lower limit of quantitation (BLQ).

^e^Unable to determine (UTD).

**Figure 3:**
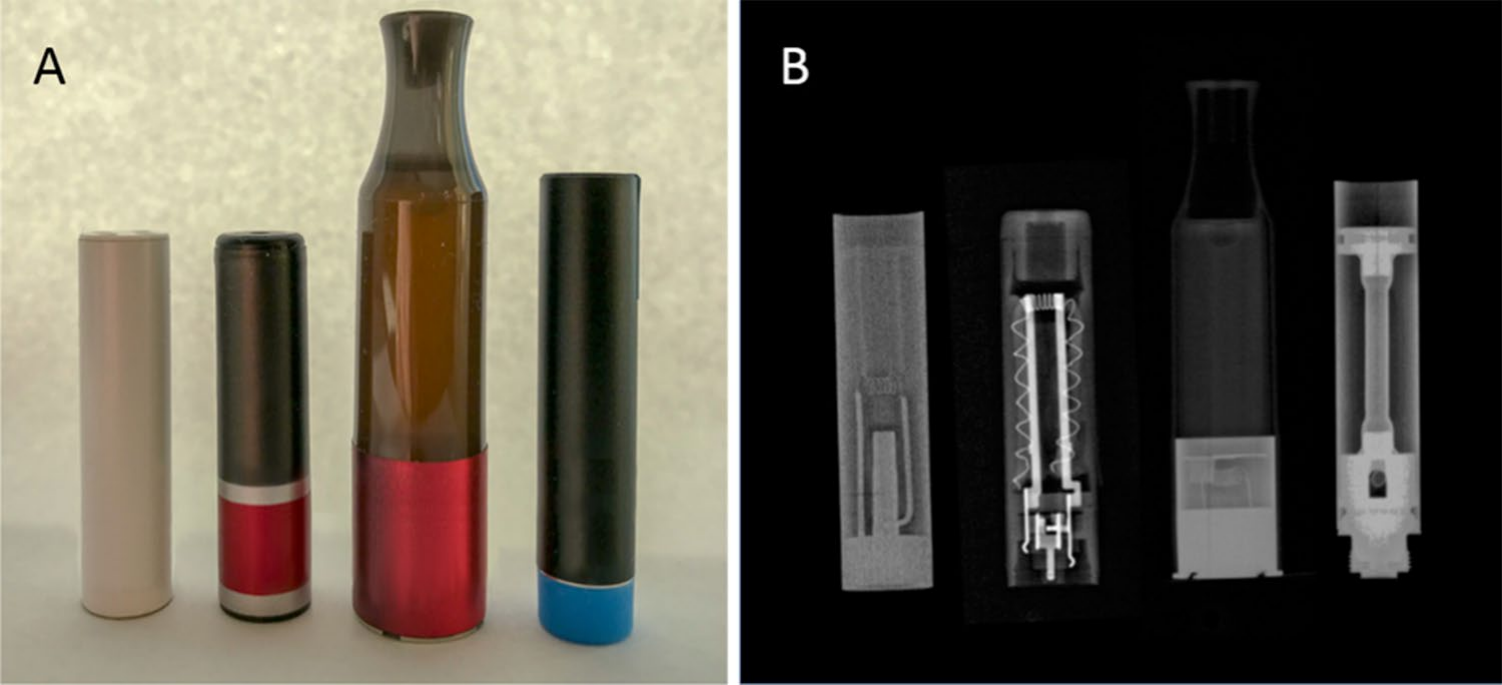
(**A**) (From left to right) Photographs of the cigalike ENDS cartridges studied: MarkTen, VUSE Solo, VUSE Vibe and blu. (**B**) (From left to right) Composite planar digital X-ray images of MarkTen, VUSE Solo, VUSE Vibe and blu e-liquid cartridges showing proximity of e-liquids to internal hardware components.

#### Metals in e-liquids: copper (Cu)

The ICP-MS data for Cu in the products purchased are presented in Table 4. The USP upper limit for Cu is 3 μg/g inhalation concentration and 300 μg/g oral concentration. USP values were exceeded in blu, Vuse Solo and Vuse Vibe, with large variation amongst the purchases (ranging from 3.6 ± 0.3 to 180 ± 20 μg/g).

**Table 4:** Concentration of copper (Cu) in µg/g of e-liquid for the purchased cigalike ENDS cartridges.

Product	Flavor	Purchases				
		Copper µg/g				
		1	2	3	4	Average
blu	Classic Tobacco	**69 (1)**^a,b^	**6.2 (0.4)**	**92 (0.1)**	**120 (9)**	**71 (40)**
	Carolina Bold	1.3 (0.1)	**8.0 (0.6)**	**30 (0.6)**	N/A^c^	**13 (10)**
	Magnificent Menthol	**120 (10)**	**59 (2)**	**23 (0.3)**	**180 (20)**	**100 (70)**
	Cherry Crush	**33 (0.5)**	N/A	1.9 (0.03)	**47 (7)**	**27 (20)**
MarkTen	Classic	0.50 (0.00)	0.66 (0.02)	0.41 (*)	0.37 (0.01)	0.51 (0.12)
	Menthol	0.66 (0.05)	BLQ^d^	BLQ	BLQ	0.66 (0.05)
	Summer Fusion	BLQ	BLQ	0.32 (*)	BLQ	0.32 (*)
	Winter Mint	BLQ	BLQ	BLQ	BLQ	UTD^e^
Vuse Solo	Original	BLQ	BLQ	**61 (20)**	**5.9 (0.9)**	**34 (30)**
	Menthol	BLQ	**43 (8)**	**15 (3)**	**12 (5)**	**23 (20)**
	Mint	BLQ	BLQ	BLQ	N/A	UTD
	Berry	BLQ	BLQ	BLQ	BLQ	UTD
	Chai	BLQ	0.56 (0.02)	BLQ	0.45 (*)	0.53 (0.06)
	Crema	BLQ	BLQ	BLQ	**66 (4)**	**66 (4)**
Vuse Vibe	Original	BLQ	BLQ	BLQ	0.94 (0.3)	0.94 (0.3)
	Menthol	N/A	BLQ	N/A	**29 (0.1)**	**29 (0.1)**
	Nectar	0.58 (0.00)	BLQ	BLQ	**5.3 (0.8)**	2.9 (3)
	Melon	BLQ	BLQ	**3.6 (0.3)**	**130 (100)**	**78 (100)**
Blank	Propylene Glycol	BLQ				
	Glycerol	0.55 (*)				

All samples for which the mean copper concentration is equal to or exceeds the USP limits for Cu (3 μg/g) are bolded.

^a^Concentrations are reported as mean (standard deviation), n = 3 unless otherwise noted by (*).

^b^Bold indicates the mean metal concentration exceeded the USP limit for an inhaled product.

^c^N/A indicates the brand and flavor combination was unavailable during the purchase attempt.

^d^Below lower limit of quantitation (BLQ).

^e^Unable to determine (UTD).

**Figure 4:**
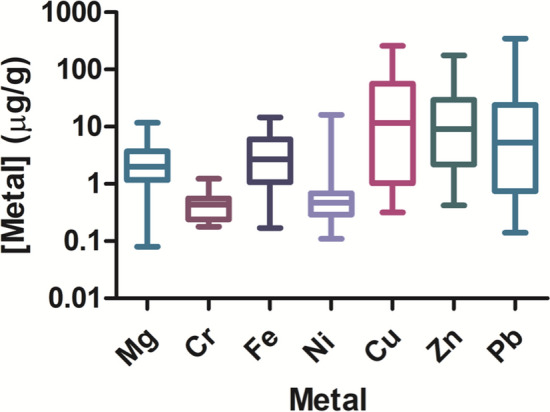
Box and whisker plot showing the range of metal concentrations found in cigalike ENDS cartridges across products. The median is shown with a horizontal line, with the box depicting the 1st–3rd quartile range. The minimum and maximum concentrations detected are shown by the whiskers.

#### Metals in e-liquids: nickel (Ni)

The ICP-MS data for Ni in the products purchased are presented in Table 5. The USP upper limit for Ni is 0.5 μg/g inhalation concentration and 20 μg/g oral concentration. USP values were exceeded in blu, Vuse Solo and Vuse Vibe, ranging from 0.52 ± 0.01 to 11 ± 5 μg/g with large variation amongst the purchases.

**Table 5:** Concentration of nickel (Ni) in µg/g of e-liquid for the purchased cigalike ENDS cartridges.

Brand	Flavor	Purchases				
		Nickel µg/g				
		1	2	3	4	Average
blu	Classic Tobacco	0.37 (0.02)^a,b^	0.43 (0.02)	0.36 (0.01)	**0.75 (0.02)**	0.48 (0.2)
	Carolina Bold	0.27 (0.02)	**0.7 (0.4)**	**1.5 (0.3)**	N/A^c^	**0.8 (0.6)**
	Magnificent Menthol	**0.63 (0.04)**	**0.65 (0.3)**	BLQ^d^	**0.66 (0.1)**	**0.65 (0.14)**
	Cherry Crush	0.29 (0.01)	N/A	0.18 (*)	0.44 (0.03)	0.34 (0.1)
MarkTen	Classic	0.28 (0.01)	0.27 (*)	0.26 (0.01)	0.15 (0.01)	0.24 (0.05)
	Menthol	BLQ	BLQ	BLQ	BLQ	UTD ^e^
	Summer Fusion	0.52 (*)	0.28 (0.01)	0.31 (0.03)	BLQ	0.33 (0.09)
	Winter Mint	0.18 (0.0)	BLQ	BLQ	0.32 (0.2)	0.25 (0.1)
Vuse Solo	Original	0.44 (0.01)	0.47 (0.0)	0.42 (0.1)	0.36 (0.1)	0.42 (0.08)
	Menthol	0.39 (0.1)	0.40 (0.02)	0.21 (*)	0.23 (0.03)	0.33 (0.1)
	Mint	**0.66 (0.04)**	**0.56 (0.01)**	0.32 (*)	N/A	0.56 (0.1)
	Berry	**0.62 (0.05)**	0.39 (0.01)	0.60 (*)	0.42 (0.01)	0.48 (0.1)
	Chai	**0.73 (0.06)**	0.47 (0.0)	**0.52 (0.01)**	**0.73 (0.2)**	**0.62 (0.2)**
	Crema	**0.69 (0.02)**	**0.96 (0.02)**	**0.64 (0.04)**	**1.06 (0.02)**	**0.82 (0.2)**
Vuse Vibe	Original	BLQ	BLQ	0.13 (0.01)	0.54 (0.08)	0.38 (0.2)
	Menthol	N/A	BLQ	N/A	**2.4 (0.5)**	**2.4 (0.5)**
	Nectar	0.11 (*)	BLQ	BLQ	**2.2 (1)**	**1.7 (1)**
	Melon	0.11 (0.0)	0.51 (*)	**6.2 (2)**	**11 (5)**	**5.1 (5)**
Blank	Propylene Glycol	BLQ				
	Glycerol	BLQ				

All samples for which the mean copper concentration is equal to or exceeds the USP limits for Ni in inhaled drug products (0.5 μg/g) are bolded.

^a^Concentrations are reported as mean (standard deviation), n = 3 unless otherwise noted by (*).

^b^Bold indicates the mean metal concentration exceeded the USP limit for an inhaled product.

^c^N/A indicates the brand and flavor combination was unavailable during the purchase attempt.

^d^Below lower limit of quantitation (BLQ).

^e^Unable to determine (UTD).

#### Additional metals in e-liquids

Four other metals: magnesium (Mg), iron (Fe), zinc (Zn) and cobalt (Co) were also detected and quantified in the e-liquids. These levels of these metals have no USP limits. Our data, shown in Supplementary Table S21–S24, show the same level of variability between purchases as those metals mentioned above.

### X-ray imaging e-cigarette hardware

We obtained composite planar digital X-ray images of one of each type of cartridge under study. As shown in Fig. 3A, B, the metal components and the heating coil are clearly visible in each product. These images provide a glimpse of the inner design to establish which surfaces in the cartridge are likely to be in contact with the e-liquid during storage.

### Metals in ENDS hardware

After X-ray imaging, each cartridge was disassembled into its component parts. The component parts were then washed to remove any residual e-liquid and surface metals were then extracted via addition of nitric acid. Subsequent ICP-MS analysis detected Pb, Cr, Cu, Ni, Mg, Fe and Zn in the extraction liquid of one or more of the cartridge components at concentrations exceeding the lower level of quantification (LLOQ) of the ICP-MS. Based upon the X-ray images, these cartridge components are likely in contact with the e-liquid, suggesting a potential source of the metals found in the e-liquids of each brand. We note that the pH of the e-liquid may influence the rate of extraction of the metals from the cartridge components, leading to higher levels of e-liquids in certain products. Similarly, the age of the product may also influence metal levels in the e-liquids, with increased levels of metals over time.

## Discussion

One key finding from these studies, in which we evaluated the e-liquids of 18 distinct ENDS products (4 different brands, 18 flavors) for metal content, is that many of the products contain levels of metals that exceed those allowed by USP in pharmaceutical products, and which can be toxic to humans (e.g. lead and chromium) (Fig. 4, Table S5). We compared the levels measured to USP limits because limits for metals in ENDS products have not yet been established.

The metals that were elevated included lead (0.27 ± 0.03 to 290 ± 50 μg/g)—up to three orders of magnitude higher than USP limits; chromium (0.31 ± 0.01 to 1.2 ± 0.05 μg/g)—up to four times as high as the USP limits; copper (3.6 ± 0.3 to 180 ± 20 μg/g) up to five times as high as the USP limits, and nickel (0.52 ± 0.01 to 11 ± 5 μg/g)—up to twenty times as high as the USP limits. There have been a handful of recent reports of elevated levels of metals in e-liquids—including in cigalike ENDS products; albeit in different brands. These include a study that just focused on measuring lead in the e-liquid of nicotine-free disposable ENDS^25^, where up to 840 ppb lead was measured; a study of a series of cigalike products (where e-liquids from each brand/same lot were combined) whereby cadmium, chromium and lead were noted to be high^26^; and a study in which the e-liquid and resultant aerosol of a series of commercially available ENDS were measured and compared to those in conventional cigarettes with multiple metals found in each type of product^13^. In this latter study, metals detected in e-liquids were also detected in the corresponding aerosols, suggesting transfer from the e-liquid, and notably more metals were present in the ENDS aerosol than in the cigarette smoke^13^. It is difficult to compare the quantities of metals measured between these emerging studies, as there is not yet a unified measure of reporting (e.g. we measure in and report micrograms per gram and some others report micrograms per liter); however collectively there is a growing body of evidence for elevated levels of metals in e-liquids.

Overall, our finding that potentially toxic metals are present in ENDS e-liquids may be of concern as these metals have the potential to transfer to the aerosol and result in user inhalation exposure. These metals—lead, chromium, copper and nickel—are known to be detrimental to human health when exposed, even at low levels. Humans do not require lead, nickel and chromium for any biological function and exposure to these metals can be harmful. Lead is a known toxicant and causes neurological damage, especially in developing brains^27,28^. In young adults, elevated lead levels are also correlated with increased depression and panic attacks^29,30^. Elevated levels of nickel and chromium are associated with decreased lung function, bronchitis, asthma in the short term, and cancer in the long term^31^. Copper is utilized by humans for multiple physiological processes including aerobic metabolism, neurotransmission and cell growth; however, elevated copper is associated with several CNS disorders^32^. On a broader scale, there are multiple epidemiological studies that link long term exposure of elevated levels of metals (often referred to as ‘trace metals’) to cancer^33,34^. As ENDS are relatively a new tobacco product category and, in some cases, resemble other consumable products (e.g. JUUL resemble flash drives), it becomes imperative to monitor the long term adverse effects on public health from ENDS use. In addition, middle and high-schoolers are increasingly using ENDS^35,36^. Some of the metals identified in the e-liquids, especially lead, are particularly detrimental to developing brains, raising particular concerns about metal exposures for this user demographic^27,28^.

We do not yet know what fraction of the metals present in the e-liquid of these cigalike products are aerosolized and inhaled by the ENDS user, perhaps augmented by metals released from the heating coil during aerosolization. There are conflicting data in the literature regarding metals in ENDS aerosols. Rule and co-workers have reported that aerosols from user-refillable tank type ENDS contain elevated levels of toxic metals (presumably from the e-liquid and hardware)^26^; whereas, Kosmider and co-workers did not detect significantly elevated levels of lead or cadmium in aerosols generated from e-liquids containing elevated levels of lead and cadmium from the same type of user-refillable tank-type ENDS^21^. Of particular relevance to our study is the work of Talbot and co-workers who showed that aerosols from disposable cigalike ENDS contain elevated levels of toxic metals^13^. The authors used the same type (but different brands) of disposable ENDS products to measure e-liquid generated aerosol metal content that we used to measure the e-liquid metal content in this study , suggesting that the toxic metals that we observed in the e-liquid of cigalike ENDS may find their way into the inhaled aerosol creating the potential for harm^15^. We also note that our approach to measure the metals in e-liquids is rapid and high-throughput, and thus has the potential to be applied to the still increasing number of e-liquids that are currently on the market, serving as a valuable resource for the scientific community engaged in ENDS related research.

A second important finding from our studies was the large variability in metal levels measured in different ‘buys’ of the same products. For example, the levels of lead in four buys of the blu “Magnificent Menthol” product were 5.3, 23, 47 and 180 μg/g. These results suggest the importance of quality control at the level of production. Currently, ENDS products are not required to adhere to any specific manufacturing standards. In addition to variability in the e-liquid, the lack of standards likely leads to variability in product hardware^37^. The implementation of quality control measures have the promise of mitigating these large variances observed in ENDS products^37^, and warrant further consideration.

Our finding that there is inter-batch variability within the same brand and flavor of cigalike ENDS products raises the question *what are the factors leading to this variability?* One factor may be the source of constituents used to formulate the different e-liquids. By purchasing ENDS over a period of 6 months, the products we obtained represented several different batch numbers, which could have contained components representing different lots, grades or vendors of ingredients. A second factor is storage conditions. The products we evaluated were purchased from gas stations (often from small kiosks between gas pumps) and convenience stores, where they were kept stacked in various orientations on shelves with no apparent temperature control. Aside from obvious product stability implications, we observed the same metals in the ENDS cartridge hardware that we detected in the e-liquid therein. A reasonable hypothesis is that the cartridge materials of construction are the source of at least some of these metals, which leach into the e-liquid over time. Irrespective of the source of the metals, for quality control, improved manufacturing practices and storage recommendations for ENDS and e-liquid formulations may be needed. For example, when metal concentrations are found to escalate over time this may warrant attribution of a *do not use after date* to the outer packaging of cigalike ENDS. We note that Nu Mark LLC, makers of MarkTen, have taken this step. Although the basis for this *do not use after date* is unclear, their products are associated with the smallest number of potentially harmful metals present at the lowest levels.

### Utility of a robust sampling approach for e-liquids in cigalike ENDS cartridges

Ultimately, validated and consistently employed approaches for e-liquid evaluation necessitates publicly available methods and standards. In the case of drug products, pharmacopoeias such as the USP often fill this role. The development of an analogous approach for ENDS has the potential to provide manufacturing guidelines and inform regulatory process and activities. The approach that we developed to sample cigalike ENDS cartridges prefilled with e-liquids by crushing, extracting e-liquid and ICP-MS analysis has the simplicity, efficiency, and availability of required materials typical of public standards, and might usefully contribute the development of future approaches to measure and potentially limit metal levels in e-liquids. The same e-liquid sampling protocol might also be useful for confirming such things as nicotine content matching the manufacturers’ label claim or that only ingredients listed on the label are present in the commercialized product. X-ray imaging has the potential to be used as a tool to ensure consistent assembly of metal components within many cigalike ENDS. This may also be a valuable tool to evaluate concerns regarding the possibility of inhaling metal fragments.

The studies presented herein are by no means comprehensive. We only examined a subset of the hundreds of ENDS products on the market; however, our approach is high-throughput and has the capability to be expanded to many more products. In addition, we measured the metal content in e-liquids and cartridge material. ENDS users inhale the aerosol, not the e-liquid, therefore we do not have a direct measure of the metals relevant to users’ exposure via aerosol. There is evidence that the metals from e-liquids can transfer to the aerosol^13^; however, transfer of the metals in the products described here to aerosol has not yet been shown. Future studies to demonstrate a connection between the metal levels in the e-liquid and the aerosol generated will be informative.

## Conclusions

The studies described herein are an important first step towards identifying and evaluating the potential harms from ENDS use. They establish that e-liquids from cigalike ENDS can contain levels of metals that exceed those acceptable for drug products, and they provide evidence that these metals may be derived from the ENDS hardware. The methodology used in this study has the potential to be applied to other ENDS products, including JUUL, which have recently received significant attention due to their increasing popularity with teens and young adults. Notably, the FDA recently issued a new guidance that will restrict the sale of ENDS that contain flavored e-liquids (other than tobacco, mint, menthol or non-flavored) to adults above age 18^38^. We envision that the evaluation of metals in e-liquids could be performed in conjunction with the evaluation of flavorings in e-liquids (which could potentially contain toxicants) to better understand how the chemical flavors might affect human health. Additionally, these studies provide the basis for future studies aimed at determining the metal content of the aerosols formed from the heated e-liquid, and the potential synergistic effects of elevated metal levels (and other potentially harmful constituents) on human health after inhalation. In short, these tools have the potential to address outstanding questions concerning appropriate specifications for ENDS, and how their use affects human health.

## Methods

### Materials

MarkTen® (Nu Mark LLC, Richmond, VA), VUSE Solo® and VUSE Vibe™ (RJ Reynolds Aerosol Company, Winston-Salem, NC), and blu® (Fontem Holdings, China) ENDS cartridges (Fig. 3A) containing e-liquids with the flavors shown in Table 1 were evaluated. Where a *do not use after date* was provided, testing was concluded before that date. USP grade propylene glycol (PG, Sigma-Aldrich, Saint Louis, MO) and glycerol (VG, Sigma-Aldrich, Saint Louis, MO), which are used alone or in combination as the vehicle in many e-liquids, were analyzed for comparison. Digital X-ray images (Fig. 3B) of the internal structure of cartridges were obtained using a Faxitron (Faxitron Bioptics, Tucson, AZ) at 60 kV for 10 s to confirm whether e-liquids were in contact with metal components.

### Sample collection: e-liquids

Extraction of the e-liquids from the sealed prefilled cartridges of each product evaluated required that a metal free method be employed. As no method was reported in the literature, we developed the following method. Each cartridge was placed in a Ziploc bag (SC. Johnson & Son, Racine, WI,) and the Ziploc bag was positioned between padded vise jaws, such that the cartridge was located at the top of the bag. The cartridge was then deformed with the vice until it ruptured, at which point the e-liquid eluted to the bottom of the Ziploc bag (shown in Fig. 1). The bag was then opened to recover the e-liquid (when necessary, with the aid of centrifugation). The e-liquid was split into three aliquots and transferred to three pre-weighted 15 mL metal free conical tubes. The tubes were then re-weighed to determine the weight of the e-liquid collected, followed by dilution to 5 mL with 6% trace metal-free nitric acid solution for ICP-MS analysis. On average, 50 mg of e-liquid was collected from each product for analysis and three independent measures were made. The e-liquid samples were digested by placing the conical tubes in an oven set to 80 °C for 12 h. Each brand and flavor combination evaluated in this study was sampled in triplicate. We note that all plastic bags, transfer pipettes, and storage vials were confirmed to be metal-free (data not shown), and all glassware were acid-washed and rinsed with Milli-Q grade water, which was also used for quantitative sample dilution.

### Sample collection: ENDS cartridge materials of construction (hardware)

Following the extraction of the e-liquid from each cartridge, the ruptured cartridges were disassembled into their component parts, some of which are visible in Fig. 3B. Due to their mechanical strength, this required the use of metal tools. The deconstructed cartridge components were extensively rinsed and sonicated in hot soapy water and then in methanol, to remove any residual e-liquid or metal fragments from the cutting tools. After a final rinse in methanol and drying, each sample was placed in trace metal grade nitric acid and left to soak for 24 h (Thermo Fisher Scientific, Waltham, MA). The nitric acid was then transferred to metal free tubes and analyzed via ICP-MS to identify the metals present in the hardware .

### Metal detection by inductively coupled plasma mass spectrometry

E-liquid metal concentrations were measured on an Agilent 7,700 × inductively coupled plasma mass spectrometer (ICP-MS) (Agilent Technologies, Santa Clara, CA), utilizing an Octopole Reaction System cell (ORS) in He mode to remove interferences^39^. The ICP-MS was warmed up and tuned per the manufacturer protocols. A typical tune performance report gave counts from 2,100 to 5,100 with RSD% 2.5–3.5 for Masses 59, 89, and 205. Typical oxide and doubly charged ratios were 0.39% and 1.3%, respectively^39^. The ICP-MS parameters used for the analysis were: an RF power of 1,550 W, an argon carrier gas flow of 0.99 L/min, helium gas flow of 4.3 mL/min, octopole RF of 200 V, and OctP bias of − 18 V^39^. Samples were directly infused using the 7700X peristaltic pump with a speed of 0.1 rps and a micromist nebulizer^39^. Metal concentrations were derived from a calibration curve generated by a dilution series of atomic absorption standards (Millipore Sigma, Saint Louis, MO) prepared in the same matrix as the samples^38^. The reported values are an average of 3 measures for each analyte of interest per sample and acid blank controls were run to insure no carry over between samples. Data analysis was performed using Agilent’s Mass Hunter software (4.4)^39^. LLOQ are as follows in parts per billion (ppb, µg/L): Mg: 2.0, Cr: 2.0, Fe: 5.0, Co: 2.0, Ni: 2.0, Cu: 5.0, Zn: 10, As: 2.0, Cd: 2.0 and Pb 2.0. For metals Mg, Cr, Fe, Co, Ni, Cu, Zn, As, Cd, and Pb by ICP-MS: the CV of within-run precision are 0.3–1.8% (0.9–5.3% for LLOQs) and inter-batch precision are 0.5–3.0% (1.5–6.0% for LLOQs); the accuracy ranged from 0.05 to 5.49% (0.14–19.4% for LLOQs) (Tables S1–S20).

### Model e-liquid percent metal recovery

Two common e-liquid bases (70% propylene glycol 30% vegetable glycerol (70 PG/ 30 VG), and 100% vegetable glycerol (100 VG)) were used for metal recovery measurements. For lead and copper samples, solutions of 1, 100, and 200 µg/g and for chromium and nickel samples, 1, 5, and 10 µg/g were prepared in each e-liquid base. Samples were then digested for 12 h at 80 °C, diluted 1:300 in 6% trace metal free nitric acid and analyzed via ICP-MS. The resultant percent recovery was between 98 and 112%, and data are shown in the supplementary materials section (Tables S25–S28).

## Supplementary information

Supplementary information.
