# Supplementary material for: Cigalike electronic nicotine delivery systems e-liquids contain variable levels of metals
Source: Sci Rep. 2020 Jul 17;10:11907. doi: 10.1038/s41598-020-67789-7 (PMC7368082; doi:10.1038/s41598-020-67789-7)
Supplement: Supplementary file 1 — Supplementary information. [file 41598_2020_67789_MOESM1_ESM.docx]

SUPPLEMENTARY INFORMATION

Cigalike Electronic Nicotine Delivery Systems E-liquids Contain Variable Levels of Metals

Heather M. Neu^a^, Angela Lee^a^, Joel E. P. Brandis^a^, Vyomesh Patel^b^, Abraham Schneider,^c^ Maureen A. Kane,^a^ Richard N. Dalby,^a^ Sarah L.J. Michel^a,*^

^a^Department of Pharmaceutical Sciences, University of Maryland School of Pharmacy, Baltimore, MD, USA

^b^Center for Tobacco Products, US Food and Drug Administration, Silver Spring, MD, USA

^c^Department of Oncology and Diagnostic Sciences, University of Maryland School of Dentistry, Baltimore, MD, USA

^*^Corresponding Author.

**ICP-MS Method Validation**

Two common e-liquid matrices were prepared: 70 % propylene glycol: 30% vegetable glycerin (70 PG/ 30 VG) and 100 % vegetable glycerin (100 VG)) using USP grade reagents (1,2, propane diol and glycerin respectively). To a 30 mL solution of 70 PG/ 30 VG in a metal-free conical tube, PbCl_2_, CuCl_2_, K_2_Cr_2_O_7_ or NiCl_2•_6H_2_O were added to final concentrations of 250 µg/g lead, 110 µg/g chromium, 75 µg/g nickel, and 280 µg/g copper. Similarly, to a 30 mL solution of 100 % VG PbCl_2_, CuCl_2_, K_2_Cr_2_O_7_ or NiCl_2•_6H_2_O were added to final concentrations of 300 µg/g lead, 95 µg/g chromium, 65 µg/g nickel, and 250 µg/g copper. The solutions were then heated for 5 hours at 80 °C followed by shaking overnight. These stock e-liquid samples were then diluted such that the metal concentrations were 1, 5, and 10 µg/g chromium or nickel in the e-liquid or 1, 100, and 200 µg/g lead and copper in the e- liquid, to reflect the range of concentrations that these metals were detected in the e-liquid from the commercial products studied. The samples were prepared for ICP-MS analysis by diluting each metal e-liquid stock in 6% trace metal free nitric acid solution to a volume of 2 mL, followed by digestion for 12 hours at 80 °C. The samples were then diluted 1:300 in 6% trace metal free nitric acid and analyzed via ICP-MS.

**ICP-MS Calibration Curve**

Calibration curves were constructed using calibration samples that were prepared fresh each day. The acceptance criterion for the calibration samples was ± 15%, except for the Lower Limit of Quantitation (LLOQ), which was ± 20% of the theoretical concentration. At least 75% of the non-zero standards were required to meet this criteria with the LLOQ passing to use the calibration curve. The acceptance criterion for the correlation coefficient of determination (R^2^) for the calibration curve was 0.99. LLOQ’s response needs to be 5 times the response of the blank.

**Table S1:** LLOQ Determination for Magnesium (Mg).

**Table S2:** Intra- and inter day data for Magnesium (Mg)

**Table S3:** LLOQ Determination for Chromium (Cr)

**Table S4:** Intra- and interday data for Chromium (Cr)

**Table S5:** LLOQ Determination for Iron (Fe)

**Table S6:** Intra- and interday data for Iron (Fe)

**Table S7:** LLOQ Determination for Cobalt (Co)

**Table S8:** Intra- and interday data for Cobalt (Co)

**Table S9:** LLOQ Determination for Nickel (Ni)

**Table S10:** Intra- and interday data for Nickel (Ni)

**Table S11:** LLOQ Determination for Copper (Cu)

**Table S12:** Intra- and interday data for Copper (Cu)

**Table S13:** LLOQ Determination for Zinc (Zn)

**Table S14:** Intra- and interday data for Zinc (Zn)

**Table S15:** LLOQ Determination for Arsenic (As)

**Table S16:** Intra- and interday data for Arsenic (As)

**Table S17:** LLOQ Determination for Cadmium (Cd)

**Table S18:** Intra- and interday data for Cadmium (Cd)

**Table S19:** LLOQ Determination for Lead (Pb)

**Table S20:** Intra- and interday data for Lead (Pb)

|  | **Intraday** | | **Interday** | |
| --- | --- | --- | --- | --- |
| **Calibration Point** | **Average (ppb)** | **% CV** | **Average (ppb)** | **% CV** |
| **2 ppb** | 2.0 (± 0.0) | 2.0 | 2.1 (± 0.1) | 2.9 |
| **5 ppb** | 5.0 (± 0.1) | 1.3 | 5.1 (± 0.1) | 2.1 |
| **10 ppb** | 11 (± 0.1) | 0.6 | 11 (± 0.2) | 1.4 |
| **50 ppb** | 52 (± 0.7) | 1.3 | 52 (± 0.8) | 1.5 |
| **100 ppb** | 100 (± 0.4) | 0.4 | 100 (± 2) | 1.9 |
| **250 ppb** | 250 (± 1) | 0.6 | 260 (± 3) | 1.3 |
| **500 ppb** | 500 (± 3) | 0.7 | 510 (± 6) | 1.1 |
| **1000 ppb** | 1000 (± 3) | 0.3 | 1,000 (± 10) | 1.3 |
| **2500 ppb** | 2,500 (± 30) | 1.2 | 2,500 (± 40) | 1.7 |

**Figure S1:** Typical calibration curve


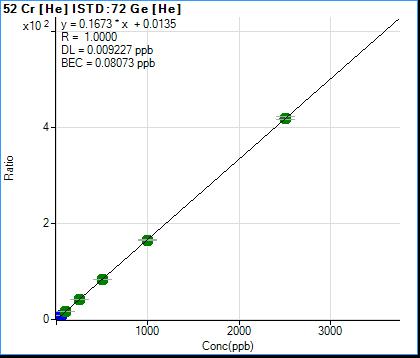


**Table S21:** ICP-MS data for Magnesium (Mg) in the products purchased

| Brand | Flavor | Purchases | | | | |
| --- | --- | --- | --- | --- | --- | --- |
|  |  | (metal concentration in µg/g of e-liquid) | | | | |
|  |  | 1 | 2 | 3 | 4 | Average |
| blu | Classic Tobacco | 11 (0.1) ^a,b^ | 8.9 (0.4) | 10 (0.08) | 11 (0.9) | 10 (0.9) |
|  | Carolina Bold | 0.61 (0.02) | 0.77 (0.01) | 0.60 (0.01) | N/A ^c^ | 0.66 (0.08) |
|  | Magnificent Menthol | 1.3 (0.02) | 1.4 (0.02) | 1.0 (0.03) | 1.1 (0.09) | 1.2 (0.2) |
|  | Cherry Crush | 2.6 (0.06) | N/A | 2.6 (0.01) | 2.7 (0.2) | 2.6 (0.1) |
| MarkTen | Classic | 3.5 (0.06) | 3.8 (0.07) | 2.8 (0.09) | 2.4 (0.09) | 3.1 (0.6) |
|  | Menthol | 1.3 (0.1) | 0.86 (0.07) | 0.68 (0.01) | 0.69 (0.1) | 0.85 (0.2) |
|  | Summer Fusion | 1.9 (0.4) | 2.9 (0.1) | 2.1 (0.1) | 1.4 (0.2) | 2.1 (0.6) |
|  | Winter Mint | 1.9 (0.1) | 1.4 (0.07) | 0.81 (0.05) | 1.1 (0.04) | 1.3 (0.5) |
| Vuse Solo | Original | 2.3 (1) | 2.2 (*) | 4.0 (*) | 1.5 (0.1) | 2.2 (1) |
|  | Menthol | BLQ^d^ | BLQ | 3.0 (*) | 1.2 (0.08) | 1.6 (0.9) |
|  | Mint | 3.0 (0.06) | 1.6 (0.04) | 3.3 (0.5) | N/A | 2.4 (0.8) |
|  | Berry | 4.0 (0.04) | 2.3 (0.2) | 4.3 (0.5) | 1.5 (0.03) | 3.0 (1) |
|  | Chai | 5.1 (1) | 1.9 (0.09) | 1.7 (*) | 2.6 (0.1) | 3.0 (2) |
|  | Crema | 4.7 (0.1) | 5.4 (0.07) | 3.9 (0.2) | 4.0 (0.08) | 4.5 (0.6) |
| Vuse Vibe | Original | 0.16 (*) | BLQ | BLQ | 0.22 (0.01) | 0.20 (0.03) |
|  | Menthol | N/A | BLQ | N/A | BLQ | UTD^e^ |
|  | Nectar | BLQ | 1.4 (*) | BLQ | 0.19 (*) | 0.77 (0.8) |
|  | Melon | 0.08 (*) | BLQ | 0.18 (*) | BLQ | 0.13 (0.07) |
| Blank | Propylene Glycol | BLQ | | | | |
|  | Vegetable Glycerin | BLQ | | | | |
| ^a^ Concentrations are reported as mean (standard deviation), n = 3 unless otherwise noted by (*) | | | | | | |
| ^b^ Bold indicates the mean metal concentration exceeded the USP limit for an inhaled product. | | | | | | |
| ^c^ N/A indicates the brand and flavor combination was unavailable during the purchase attempt.  ^d^ Below lower limit of quantitation (BLQ)  ^e^ Unable to determine (UTD) | | | | | | |

**Table S22:** ICP-MS data for Iron (Fe) in the products purchased

| Brand | Flavor | Purchases | | | | |
| --- | --- | --- | --- | --- | --- | --- |
|  |  | (metal concentration in µg/g of e-liquid) | | | | |
|  |  | 1 | 2 | 3 | 4 | Average |
| blu | Classic Tobacco | 8.0 (0.2) ^a,b^ | 11 (0.5) | 5.5 (0.5) | 10 (0.9) | 8.6 (2) |
|  | Carolina Bold | 2.2 (0.2) | 1.1 (0.06) | 2.4 (0.2) | N/A ^c^ | 1.9 (0.6) |
|  | Magnificent Menthol | 11 (0.2) | 4.3 (3) | 1.3 (0.08) | 5.9 (0.5) | 5.7 (4) |
|  | Cherry Crush | 5.0 (0.1) | N/A | 1.8 (0.06) | 8.8 (0.6) | 5.2 (3) |
| MarkTen | Classic | 4.0 (0.08) | 4.1 (0.2) | 2.7 (0.01) | 1.7 (0.07) | 3.1 (1) |
|  | Menthol | BLQ ^d^ | 1.7 (0.7) | 0.41 (*) | BLQ | 1.3 (0.9) |
|  | Summer Fusion | 6.9 (*) | 4.1 (0.3) | 4.4 (1) | 1.9 (0.03) | 3.8 (2) |
|  | Winter Mint | 1.3 (0.02) | 1.5 (0.3) | 0.59 (0.02) | 1.0 (0.1) | 1.2 (0.4) |
| Vuse Solo | Original | 0.80 (0.2) | 1.4 (0.3) | 4.2 (*) | 0.60 (0.00) | 1.3 (1) |
|  | Menthol | 0.57 (*) | 1.7 (0.5) | 0.86 (0.04) | 0 (0) | 1.3 (0.6) |
|  | Mint | 9.1 (3) | 8.2 (0.1) | 3.5 (0.2) | N/A | 7.0 (3) |
|  | Berry | 8.1 (0.3) | 6.8 (0.1) | 6.7 (1) | 4.5 (0.2) | 6.5 (1) |
|  | Chai | 6.3 (*) | 5.1 (0.6) | 4.0 (*) | 6.6 (0.02) | 5.6 (1) |
|  | Crema | 6.2 (0.2) | 12.7 (2) | 5.9 (0.4) | 1.2 (0.08) | 6.5 (4) |
| Vuse Vibe | Original | 2.3 (*) | 0.61 (0.2) | BLQ | BLQ | 1.2 (1) |
|  | Menthol | N/A | 0.62 (0.09) | N/A | 0.73 (0.09) | 0.68 (0.1) |
|  | Nectar | 0.37 (0.01) | 0.66 (0.07) | 0.17 (*) | 0.74 (0.1) | 0.54 (0.2) |
|  | Melon | 0.32 (*) | 0.70 (0.01) | 2.6 (0.3) | 2.2 (1) | 1.6 (1) |
| Blank | Propylene Glycol | 0.26 (*) | | | | |
|  | Vegetable Glycerin | BLQ | | | | |
| ^a^ Concentrations are reported as mean (standard deviation), n = 3 unless otherwise noted by (*). | | | | | | |
| ^b^ Bold indicates the mean metal concentration exceeded the USP limit for an inhaled product. | | | | | | |
| ^c^ N/A indicates the brand and flavor combination was unavailable during the purchase attempt.  ^d^ Below lower limit of quantitation (BLQ) | | | | | | |

**Table S23:** ICP-MS data for Zinc (Zn) in the products purchased

| Brand | Flavor | Purchases | | | | |
| --- | --- | --- | --- | --- | --- | --- |
|  |  | (metal concentration in µg/g of e-liquid) | | | | |
|  |  | 1 | 2 | 3 | 4 | Average |
| blu | Classic Tobacco | 38 (0.7) ^a,b^ | 9.6 (0.6) | 46 (0.2) | 64 (4) | 39 (2) |
|  | Carolina Bold | 0 (0) | 3.2 (0.5) | 7.8 (0.3) | N/A ^c^ | 5.6 (3) |
|  | Magnificent Menthol | 78 (5) | 33 (2) | 14 (0.2) | 99 (9) | 58 (40) |
|  | Cherry Crush | 18 (0.4) | N/A | 2.5 (0.1) | 24 (3) | 15 (10) |
| MarkTen | Classic | 1.6 (0.01) | 2.1 (0.05) | 0.68 (*) | 0.98 (0.1) | 1.5 (0.5) |
|  | Menthol | BLQ^d^ | BLQ | BLQ | BLQ | UTD^e^ |
|  | Summer Fusion | 3.0 (*) | BLQ | 1.0 (0.2) | BLQ | 1.7 () |
|  | Winter Mint | BLQ | BLQ | BLQ | 2.3 (*) | 2.3 (*) |
| Vuse Solo | Original | BLQ | BLQ | BLQ | 2.5 (*) | 2.5 (*) |
|  | Menthol | BLQ | BLQ | BLQ | BLQ | UTD |
|  | Mint | BLQ | BLQ | BLQ | N/A | UTD |
|  | Berry | BLQ | BLQ | BLQ | BLQ | UTD |
|  | Chai | BLQ | BLQ | BLQ | BLQ | UTD |
|  | Crema | BLQ | BLQ | BLQ | BLQ | UTD |
| Vuse Vibe | Original | BLQ | BLQ | BLQ | 1.1 (0.1) | 1.1 (0.1) |
|  | Menthol | BLQ | BLQ | N/A | 25 (0.3) | 25 (0.3) |
|  | Nectar | 4.0 (0.03) | BLQ | 2.1 (0.01) | 11 (2) | 5.6 (4) |
|  | Melon | 0.42 (0) | BLQ | 9.5 (0.3) | 87 (90) | 40 (70) |
| Blank | Propylene Glycol | BLD | | | | |
|  | Vegetable Glycerin | 0.14 (0.2) | | | | |
| ^a^ Concentrations are reported as mean (standard deviation), n = 3 unless otherwise noted by (*). | | | | | | |
| ^b^ Bold indicates the mean metal concentration exceeded the USP limit for an inhaled product. | | | | | | |
| ^c^ N/A indicates the brand and flavor combination was unavailable during the purchase attempt.  ^d^ Below lower limit of quantitation (BLQ)  ^e^ Unable to determine (UTD) | | | | | | |

**Table S24:** ICP-MS data for Cobalt (Co) in the products purchased

| Brand | Flavor | Purchases | | | | |
| --- | --- | --- | --- | --- | --- | --- |
|  |  | (metal concentration in µg/g of e-liquid) | | | | |
|  |  | 1 | 2 | 3 | 4 | Average |
| blu | Classic Tobacco | 0.01 (0) ^a,b^ | 0.01 (0) | BLD^d^ | BLD | 0.01 (0) |
|  | Carolina Bold | BLQ | BLQ | BLQ | N/A ^c^ | UTD^e^ |
|  | Magnificent Menthol | 0.01 (0) | 0.04 (0.01) | BLQ | BLQ | 0.01 (0.02) |
|  | Cherry Crush | 0.01 (0) | N/A | BLQ | BLQ | 0.01 (0) |
| MarkTen | Classic | 0.06 (0) | 0.01 (0) | 0.02 (0) | BLQ | 0.03 (0.02) |
|  | Menthol | BLQ | BLQ | BLQ | BLQ | BLQ |
|  | Summer Fusion | 0.02 (*) | 0.02 (0) | 0.01 (0) | BLQ | 0.02 (0.01) |
|  | Winter Mint | 0.01 (0) | BLQ | BLQ | BLQ | 0.01 (0.01) |
| Vuse Solo | Original | BLQ | BLQ | BLQ | BLQ | BLQ |
|  | Menthol | BLQ | BLQ | BLQ | BLQ | BLQ |
|  | Mint | 0.02 (0.01) | BLQ | BLQ | N/A | 0.02 (0) |
|  | Berry | 0.02 (0) | 0.01 (*) | 0.01 (*) | BLQ | 0.01 (0) |
|  | Chai | 0.01 (0) | 0.01 (*) | BLQ | BLQ | 0.01 (0) |
|  | Crema | 0.02 (0) | 0.02 (*) | BLQ | BLQ | 0.02 (0) |
| Vuse Vibe | Original | BLQ | BLQ | BLQ | BLQ | UTD |
|  | Menthol | N/A | BLQ | N/A | BLQ | UTD |
|  | Nectar | BLQ | BLQ | BLQ | BLQ | UTD |
|  | Melon | BLQ | BLQ | BLQ | BLQ | UTD |
| Blank | Propylene Glycol | BLQ | | | | |
|  | Vegetable Glycerin | BLQ | | | | |
| ^a^ Concentrations are reported as mean (standard deviation), n = 3 unless otherwise noted by (*). | | | | | | |
| ^b^ Bold indicates the mean metal concentration exceeded the USP limit for an inhaled product. | | | | | | |
| ^c^ N/A indicates the brand and flavor combination was unavailable during the purchase attempt.  ^d^ Below lower limit of quantitation (BLQ)  ^e^ Unable to determine (UTD) | | | | | | |

**Table S25.** The percent recovery and measured lead (Pb) concentrations in µg/g of both 70 / 30 percent propylene glycol (PG) / vegetable glycerin (VG) and 100 percent VG lead spiked e-liquids (1, 100, and 200 µg/g Pb) measured by ICP-MS. For all samples, the mean lead concentration is within 7 percent of the expected concentration and percent CV are within 6.0 percent.

| E-liquid percent composition | Metal | Expected lead µg/g | Measured lead µg/g | Percent recovery |
| --- | --- | --- | --- | --- |
| 70 PG / 30 VG | Lead | 1.0 | 1.1 (0.05) ^a^ | 107 |
|  |  | 100 | 100 (0.9) | 104 |
|  |  | 200 | 210 (1) | 103 |
|  | Blank | 0 | BLQ ^b^ | UTD ^c^ |
| 100 VG | Lead | 1.0 | 1.0 (0.06) | 100 |
|  |  | 100 | 100 (0.4) | 100 |
|  |  | 200 | 200 (2) | 98 |
|  | Blank | 0 | BLQ | UTD |
| ^a^ Concentrations are reported as mean (standard deviation), n = 5  ^b^ Below lower limit of quantitation (BLQ)  ^c^ Unable to determine (UTD) | | | | |

**Table S26.** The percent recovery and measured chromium (Cr) concentrations in µg/g of both 70 / 30 percent propylene glycol (PG) / vegetable glycerin (VG) and 100 percent VG chromium spiked e-liquids (1, 5, and 10 µg/g Cr) measured by ICP-MS. For all samples, the mean chromium concentration is within 12 percent of the expected concentration and the percent CV are within 7.4 percent.

| E-liquid percent composition | Metal | Expected chromium µg/g | Measured chromium µg/g | Percent recovery |
| --- | --- | --- | --- | --- |
| 70 PG / 30 VG | Chromium | 1.0 | 1.1 (0.04) ^a^ | 112 |
|  |  | 5.0 | 5.2 (0.06) | 103 |
|  |  | 10 | 11 (0.5) | 106 |
|  | Blank | 0 | BLQ ^b^ | UTD ^c^ |
| 100 VG | Chromium | 1.0 | 1.1 (0.08) | 108 |
|  |  | 5.0 | 5.2 (0.1) | 104 |
|  |  | 10 | 10 (0.1) | 102 |
|  | Blank | 0 | BLQ | UTD |
| ^a^ Concentrations are reported as mean (standard deviation), n = 5  ^b^ Below limit lower limit of quantitation (BLQ)  ^c^ Unable to determine (UTD) | | | | |

**Table S27** The percent recovery and measured copper (Cu) concentrations in µg/g of both 70 / 30 percent propylene glycol (PG) / vegetable glycerin (VG) and 100 percent VG copper spiked e-liquids (1, 100, and 200 µg/g Cu) measured by ICP-MS. For all samples, the mean copper concentration is within 5 percent of the expected concentration and the percent CV are within 8.2 percent.

| E-liquid percent composition | Metal | Expected copper µg/g | Measured copper µg/g | Percent recovery |
| --- | --- | --- | --- | --- |
| 70 PG / 30 VG | Copper | 1.0 | 1.02 (0.04) ^a^ | 102 |
|  |  | 100 | 105 (1) | 105 |
|  |  | 200 | 207 (7) | 104 |
|  | Blank | 0 | BLQ ^b^ | UTD ^c^ |
| 100 VG | Copper | 1.0 | 0.98 (0.08) | 98 |
|  |  | 100 | 100 (0.6) | 100 |
|  |  | 200 | 200 (3) | 100 |
|  | Blank | 0 | BLQ | UTD |
| ^a^ Concentrations are reported as mean (standard deviation), n = 5  ^b^ Below lower limit of quantitation (BLQ)  ^c^ Unable to determine (UTD) | | | | |

**Table S28.** The percent recovery and measured nickel (Ni) concentrations in µg/g of both 70 / 30 percent propylene glycol (PG) / vegetable glycerin (VG) and 100 percent VG nickel spiked e-liquids (1, 5, and 10 µg/g Ni) measured by ICP-MS. For all samples, the mean nickel concentration is within 8 percent of the expected concentration and the percent CV are within 4.8 percent.

| E-liquid percent composition | Metal | Expected nickel µg/g | Measured nickel µg/g | Percent recovery |
| --- | --- | --- | --- | --- |
| 70 PG / 30 VG | Nickel | 1.0 | 1.1 (0.04) ^a^ | 108 |
|  |  | 5.0 | 5.2 (0.04) | 104 |
|  |  | 10 | 10 (0.3) | 104 |
|  | Blank | 0 | BLQ^b^ | UTD ^c^ |
| 100 VG | Nickel | 1.0 | 1.1 (0.01) | 107 |
|  |  | 5.0 | 5.2 (0.05) | 104 |
|  |  | 10 | 11 (0.5) | 106 |
|  | Blank | 0 | BLQ | UTD |
| ^a^ Concentrations are reported as mean (standard deviation), n = 5  ^b^ Below lower limit of quantitation (BLQ)  ^c^ Unable to determine (UTD) | | | | |
